# Fluid Extract of Male Fern in Tape Worm

**Published:** 1873-06

**Authors:** 


### Fluid Extract of Male Fern in Tape Worm.

To secure the successful destruction and expulsion of tape worm, two point are to be particularly carried out. First, the patient must fast at least twelwe hours before taking the remedy; and second, it must be taken in sufficient quantities to kill and expel the entire worm. Frequently it is a matter of good policy to give the patient a cathartic in the night, so as to have the alimentary tract free from fæces as much as possible. Then in the morning, on a fasting stomach, give the male fern in some pleasant combination, as the syrup of acacia or glycerine. From thirty to sixty minims of the fluid extract of male fern, must be combined at each dose, and repeated every two hours, until the stomach rebels against it, the patient keeping very quiet in the meanwhile. No worm can resist this treatment when carried out on the above principles. The fern will move the bowels and expel the entire worm. It is the most reliable remedy for tape worm, when given in accordance with the above directions. The patient must fast during the time he is taking the remedy, and the bowels must be previously well cleared out.—*St. Louis Med. Archives.*

